# Recommendations and Alerting for Delirium Alleviation in Real-Time (RADAR): Protocol for a pilot randomized controlled trial

**DOI:** 10.12688/f1000research.20597.2

**Published:** 2020-09-01

**Authors:** Phillip E. Vlisides, Jacqueline W. Ragheb, Aleda Leis, Amanda Schoettinger, Kim Hickey, Amy McKinney, Joseph Brooks, Mackenzie Zierau, Alexandra Norcott, Shirley Yang, Michael S. Avidan, Lillian Min

**Affiliations:** 1 Department of Anesthesiology, University of Michigan Medical School, Ann Arbor, MI, 48170, USA; 2 Center for Consciousness Science, University of Michigan Medical School, Ann Arbor, MI, 48109, USA; 3 Department of Social Work, Michigan Medicine, Ann Arbor, MI, 48109, USA; 4 University of Michigan School of Nursing, Ann Arbor, MI, 48109, USA; 5 Department of Internal Medicine, Division of Geriatric and Palliative Medicine, University of Michigan Medical School, Ann Arbor, MI, 48109, USA; 6 Department of Internal Medicine, Division of Geriatric and Palliative Medicine, Veterans Affairs Ann Arbor Healthcare System, Ann Arbor, MI, 48105, USA; 7 Department of Anesthesiology, Washington University School of Medicine, St. Louis, MO, 63110, USA; 8 Geriatric Research Education and Clinical Care, VA Ann Arbor Healthcare System, Ann Arbor, MI, 48105, USA

**Keywords:** Clinical Trial Protocol, Decision Support Systems, Delirium, Feasibility Studies, Perioperative Care

## Abstract

**Background: **Delirium is a common and serious complication of major surgery for older adults. Postoperative social and behavioral support (e.g., early mobilization, mealtime assistance) may reduce the incidence and impact of delirium, and these efforts are possible with proactive patient-care programs. This pilot trial tests the hypothesis that a multicomponent decision support system, which sends automated alerts and recommendations to patient-care programs and family members for high-risk patients, will improve the postoperative environment for neurocognitive and clinical recovery.

**Methods: **This will be a randomized, controlled, factorial pilot trial at a large academic medical center. High-risk, non-cardiac surgery patients (≥70 years old) will be recruited. Patients will be allocated to a usual care group (n=15), Hospital Elder Life Program (HELP)-based paging system (n=15), family-based paging system (n=15), or combined HELP- and family-based system (n=15). The primary outcome will be the presence of delirium, defined by positive long-form Confusion Assessment Method screening. Secondary outcomes will include additional HELP- and family-based performance metrics along with various neurocognitive and clinical recovery measures. Exploratory outcomes include the incidence of positive family-based delirium assessments post-discharge, 36-item Short Form Survey, PROMIS Cognitive Function Abilities Subset 4a, and 30-day readmission rates.

**Ethics and dissemination: **This trial has received approval by the University of Michigan Medical Institutional Review Board (IRBMED). Dissemination plans include presentation at scientific conferences, publication in medical journals, and distribution via educational and news media.

**Registration: **ClinicalTrials.gov Identifier NCT04007523, registered on 7/3/2019.

## Introduction

Delirium is a distressing and common surgical complication, affecting approximately 20–50% of older surgical patients ^1, 2^. Postoperative delirium is associated with increased mortality ^3^ and cognitive and functional decline ^4– 6^, and healthcare resource utilization ^7, 8^. Of the diverse prevention strategies that have been tested with variable success ^7, 9^, one notable proactive patient-care program, the Hospital Elder Life Program (HELP), has been shown to reduce delirium incidence through social and behavioral interventions (e.g., mealtime assistance, support with visual/hearing aids) ^10^. However, substantial resources are needed for program sustainment, and delirium still persists in high-risk patients ^11– 13^. In 2018, we found that <50% of surgical patients ≥70 years old at Michigan Medicine were officially enrolled in the program by the end of the second postoperative day. Furthermore, the average length of cumulative therapeutic activity was only 10 minutes across the first three postoperative days. This is pertinent given that the peak incidence of postoperative delirium occurs within the first 48 hours ^2, 14^. As such, complementary strategies that improve patient triage and support may lead to earlier identification and therapeutic intervention for high-risk patients.

Clinical decision support systems can serve as a candidate strategy for mitigating delirium risk. Such systems provide targeted patient- and disease-specific information, presented in a timely manner, for improving healthcare quality ^15, 16^. In the context of delirium, automated pages could be sent to supportive healthcare services, such as HELP, along with family members and caretakers, with alerts and targeted recommendations. An alert page could be sent to HELP program officials on the first postoperative morning requesting early evaluation and enhanced treatment protocols. This may improve high-risk patient triage, early resource allocation, and cumulative therapeutic time spent with patients. A similar paging system could be implemented for family members and caretakers, as family-based interventions may provide additional support for patients at risk for delirium. Feasibility has been demonstrated with family-based protocols for hospitalized medical patients, with therapeutic focus on re-orientation, visual and hearing aid assistance, and conversational stimulation ^17^. Similar protocols could be adapted for surgical patients, as surgery is generally a predictable event (and thus possibly amenable to familial planning), and family support may correlate with overall postoperative recovery ^18^. A recent systematic review also demonstrated that *family-performed* delirium screens demonstrated improved psychometrics compared to *family-informed* delirium screens (i.e., those not performed by family members) ^19^. Thus, family members and caretakers could be recruited to actively participate in postoperative recovery by performing family-based delirium assessments ^20^ and implementing therapeutic protocols. An electronic, paging-based alerting system could provide family members with reminders and alerts for conducting such a program.

The premise of this pilot proposal is thus formed by the above considerations: preliminary evidence that suggests (1) suboptimal delirium prevention resource utilization and (2) the potential role for a clinical decision support system involving HELP and family members. The primary objective of this study is to determine whether pager-based clinical decision support systems enhance HELP- and family-based therapeutic activities. A secondary objective will be to identify facilitators and barriers to delivering therapeutic interventions for both HELP and family members. Overall, this pilot trial will test the hypothesis that a multicomponent decision support system will improve the postoperative environment for neurocognitive and clinical recovery in older, high-risk surgical patients.

## Methods and analysis

### Study overview and design

This is a single-center, randomized, factorial pilot trial at Michigan Medicine (Ann Arbor MI, USA). Approval was obtained from the University of Michigan Medical School Institutional Review Board (HUM00165251), and the trial has been registered at www.clinicaltrials.gov ( NCT04007523). This protocol is also compliant with the Consolidated Standards of Reporting Trials (CONSORT) extension for pilot and feasibility trials and the Standard Protocol Items: Recommendations for Interventional Trials (SPIRIT) Guidelines ^21, 22^. Lastly, all study team members are certified in Good Clinical Practice, and the study team will follow institutional protocols for conducting clinical research during the COVID-19 pandemic.

**Figure 1.**
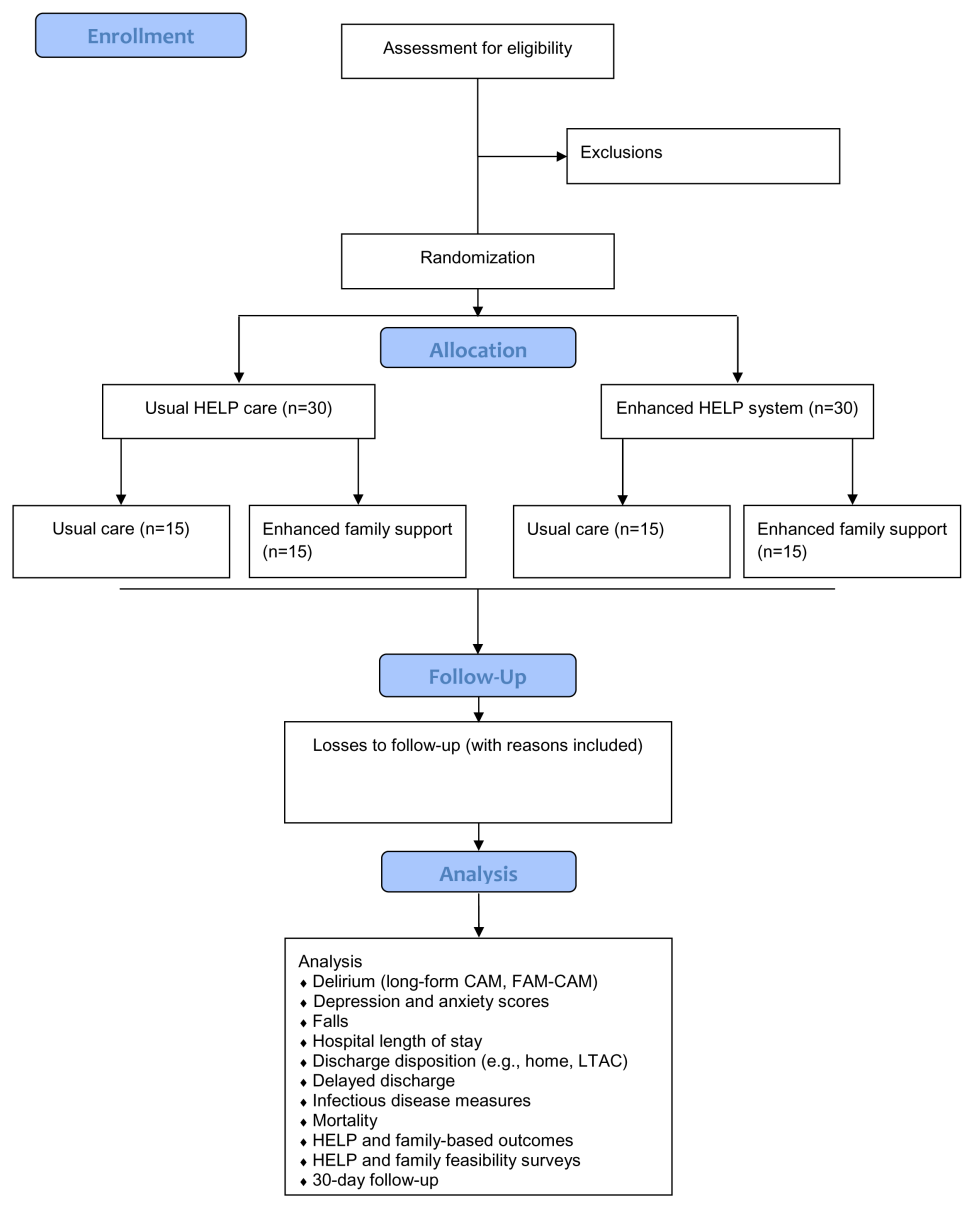
CONSORT study flow diagram. HELP = Hospital Elder Life program; CAM = Confusion Assessment Method, LTAC = Long-Term Acute Care.

After enrollment, patients (n=60) will be allocated (1:1:1:1), via block-randomization, stratified by gender, to one of four groups: usual care (n=15), HELP-based paging system (n=15), family-based paging system (n=15), or both HELP- and family-based paging system (n=15) ( Figure 1). The randomization code will be created by the biostatistician (AL) and concealed from the rest of the research team. On the morning of surgery, allocation assignments will be delivered via sequentially numbered, opaque, sealed envelopes to unblinded research team members who will initiate arm-specific operations. The support systems will consist of automated pager alerts to the HELP program and/or family members and caretakers, depending on group allocation, for providing additional delirium evaluation and therapeutic prevention activities (see *Interventions: clinical decision support systems*). Family members in the intervention group will also be provided with preoperative education on delirium and training in the Family Confusion Assessment Method (FAM-CAM) instrument ^20^. Although it will not be possible to blind patients and family members to family-based interventions, study team members performing daily assessments will remain blinded to group allocation.

### Participants

Participants will be screened and recruited at preoperative clinics, preoperative holding areas, and surgical wards (if patients are pre-admitted). Written informed consent will be obtained from all participants and family members (or caretakers) prior to scheduled surgery. Template forms are provided as *Extended data* ^23^. Supplemental recruitment materials will be distributed in conjunction with the Michigan Institute for Clinical and Health Research, the NIH-funded Clinical and Translational Science Award Institute at the University of Michigan. Specifically, recruitment fliers will be posted throughout preoperative clinics, and informative postcards will be sent to potentially eligible patients preoperatively.

Eligibility criteria will reflect the pragmatic nature of the trial balanced with the aim of recruiting patients at high risk for postoperative delirium. Based on a validated geriatric assessment tool for predicting postoperative complications, surgical patients ≥ 70 years of age presenting for major inpatient surgery demonstrated a seven-fold increased risk of major complications, including delirium, compared to minor surgery ^24^. Inclusion criteria will thus include the following: age ≥ 70 years of age; major non-cardiac, non-intracranial neurologic, and non-major vascular surgery (as defined by work-related value units suggestive of high surgical complexity), anticipated length of hospital stay at least 72 hours, and at least one family member, or caretaker, available on each of the three first postoperative days. Exclusions include emergency surgery, severe cognitive impairment (precluding ability to perform delirium assessments), planned post-operative ICU admission (HELP unavailable in the ICU), and non-English speaking.

### Interventions: clinical decision support systems

This proposal will build upon previous decision support systems launched by our department for reducing intraoperative awareness and delivering protective lung ventilation strategies ^25, 26^. For participants randomized to the HELP-based support system, a single page will be sent to the on-call HELP staff during the first postoperative morning as the team begins ward rounds ( Table 1). The page will request an enhanced treatment protocol, which includes HELP visitations three times daily. Therapeutic treatment will be administered during each visit per program protocols, which generally includes cognitive engagement, mealtime assistance, mobility and range of motion exercises, and assistance with visual and hearing aids. During the final evening visit, a sleep protocol will be implemented. For this protocol, HELP officials offer sleep and relaxation exercises, relaxation massages, and warm milk and/or tea. For the usual care group, HELP volunteers will review surgical ward censuses as able, and patients will be seen based on volunteer availability and visitation patterns. No structured, triage system will be implemented.

**Table 1.** Real-time clinical decision support – family paging system.

Days	Timing	Alphanumeric Paging Message
**HELP-Based Paging System**		
Postoperative day 1	Morning – 9:00 AM	*Patient LAST NAME, FIRST NAME, MRN is at high-risk for postoperative delirium. Please* *evaluate patient as soon as possible and enroll in the enhanced treatment protocol.*
**Family-Based Paging System**		
Postoperative days 1-3	Morning – 9:00 AM	*Good morning! Please complete the morning tasks listed in your folder (Morning Tasks* *9:00 AM). The stimulating activity can then be performed anytime during the day. Call* *734-647-8129 with questions or concerns.*
Postoperative days 1-3	Afternoon – 3:00PM	*Good afternoon! Please complete the afternoon tasks in your folder (Afternoon Tasks –* *3:00 PM). After these are complete, perform a FAM-CAM. Make sure to also complete a* *stimulating activity today. Call 734-647-8129 with any questions.*

FAM-CAM = Family Confusion Assessment Method. MRN = Medical Record Number

For participants randomized to the family-based system, family members (or caretakers) will receive preoperative education on delirium (including an educational video), a folder with an informational flyer and therapeutic activities checklists, and FAM-CAM training. Suggested therapeutic activities include daily assistance with visual and hearing aids, providing assistance with drinking and mealtime assistance, handwashing, re-orientation to time and place, and cognitive stimulation activities. Lastly, family members will also receive a pager, and automated pages will be sent twice daily with reminders to perform these activities ( Table 1). Completion of activities will then be logged daily in conjunction with unblinded members of the research team. Study activities for each group are listed in Table 2.

**Table 2.** Study operations across groups.

Usual Care Group (n=15)	HELP-Based System (n=15)	Family-Based System (n=15)	Combined Systems (n=15)
*Preoperative phase*			
Standard Care	Standard Care	Preoperative delirium education, materials, FAM-CAM training	Preoperative delirium education, materials, FAM-CAM training
*Intraoperative phase*			
Standard Care	Standard Care	Standard Care	Standard Care
*Postoperative care*			
HELP evaluation and treatment per ward routine	HELP pager alerts	Daily family pager alerts	HELP pager alerts and associated activities
Standard care otherwise	Early evaluation request	FAM-CAM	Family pager alerts and associated activities
	Enhanced therapeutic protocol request	Family-based behavioral/social support and prevention activities	

HELP = Hospital Elder Life Program; FAM-CAM = Family Confusion Assessment Method.

### Intervention fidelity

During this pilot phase, characterizing the success and barriers encountered with trial interventions will be essential for analyzing fidelity. As a separate, but complimentary line of investigation, facilitators and barriers to support system implementation will be characterized for both HELP personnel and family members. The following strategies for characterizing implementation efforts are driven by the Consolidated Framework for Implementation Research (CFIR) ^27^, which described five major domains that shape implementation effectiveness: intervention characteristics, outer setting, inner setting, characteristics of individuals involved, and the process of implementation. Survey-based questions and focus groups described below are guided by these implementation themes.

HELP-based implementation barriers will be elucidated via combination of focus groups and online surveys. This strategy has been previously used for successfully identifying facilitators and barriers to delirium prevention involving multidisciplinary bundles ^28^. Prior to paging system implementation, an anonymous survey will be distributed to HELP staff members. The survey includes Likert-scale ^29^ questions derived from the “Safety Attitudes Questionnaire,” which reports views on teamwork, safety, collaboration, resource availability, and collegiality ^30^. Open-ended questions are then provided for participants to express additional thoughts and insights. These surveys will be sent again 6 and 12 months after system implementation. Within a month after each of these surveys are collected, focus groups will be held with available HELP team members. Focus groups will be tape recorded and common themes will be elicited from transcriptions ^28, 31^. All responses will remain anonymous from both groups and surveys. The final objective will be to delineate clear barriers and facilitators to HELP-based triage and therapy implementation strategies.

All family members will be provided with surveys (available as *Extended data*) ^23^ on postoperative day three (or discharge, whichever is sooner). These surveys also contain a similar combination of Likert-scale and open-ended questions to identify barriers to completing delirium screening and prevention activities.

Lastly, a sensitivity analysis will be performed, which will report daily proportions – and reasons – for missing HELP- and family-based assessments (see Sensitivity Analysis and Missing Data).

### Outcomes

The primary outcome of this pilot trial will be the presence of delirium, defined by a positive long-form Confusion Assessment Method (CAM) ^32^ screening. The following secondary outcomes will also be collected and analyzed: delirium severity (long-form CAM severity scale), new symptoms of depression or anxiety (using the Hospitalized Anxiety and Depression Scale, HADS) ^33^, falls (proportion, %), length of hospital stay (days), discharge disposition (e.g., home, long-term care facility), delayed discharge due to cognitive impairment (proportion, %), incidence of any new non-surgical site infection (%), incidence of new multidrug resistant organism colonization (%), and mortality (%). Exploratory outcomes will include the incidence of positive FAM-CAM assessments (%) 30 days post-discharge, PROMIS Cognitive Function Abilities (Short Form 4a), 36-Item Short Form Survey, and 30-day readmission rates.

***Protocol fidelity measures.*** Lastly, protocol fidelity measures will be reported for both HELP- and family-based interventions. HELP-based measures include the following: total therapeutic time spent with HELP staff during the first three postoperative days, proportion of participants visited and enrolled by HELP (%), and time to initial HELP evaluation. For family-based interventions, the following measures will be reported: cumulative time family members spent with patients, proportion of daily tasks (e.g., assistance with glasses/hearing aids, handwashing), successfully completed, length of time spent on stimulating activity, and overall agreement of the FAM-CAM with interview-rated CAM assessments.

***Data collection.*** At Michigan Medicine, HELP data collection is standard throughout surgical and medical wards. The time at which patients are first evaluated, total therapeutic time (minutes) spent with patients, and nature of therapeutic activities (e.g., cognitive stimulation, mealtime assistance) are all collected daily and logged on computer files. Unblinded research team will have access to these logs via secured, shared drive within the Michigan Medicine network. These research personnel will review HELP logs daily and meet with HELP leadership as needed to discuss problems that may arise regarding HELP data collection and logging.

For delirium assessment, research team members will screen for delirium using the long-form CAM ^32^ twice daily (once in the morning, and again in the afternoon) for the first three postoperative days. These team members will be blinded to group allocation. Our research group has extensive experience with CAM in prior trials ^2, 34, 35^, and our international group has created a program for training investigators in CAM methodology with a previously high inter-rater reliability (Fleiss kappa=0.88 [95% CI 0.85 to 0.92]) ^35^. Our study team members who have previously received this training will lead CAM assessment efforts for this trial. Additionally, the study PI (Vlisides), has received complementary CAM training from the NIH-funded (K07AG041835) Center of Excellence for Delirium in Aging: Research, Training and Educational Enhancement (CEDARTREE). For new team members not previously trained, the PI will lead an on-site training session using online long-form CAM training videos available from the Hospital Elder Life Program. Then, after each team member has successfully scored two non-delirious and two delirious patients identically – in terms of symptom recognition – with a previously trained study team member, the trainees will be considered independently trained for CAM assessment ^2^. For those enrolled in the intervention bundle, family members (or caretakers) will perform the FAM-CAM ^20^ independently of the research team. FAM-CAM assessments will be requested once daily in the afternoon.

Depression and anxiety measures will take place both at preoperative baseline and during postoperative day three (or day of discharge, whichever is sooner). For assessment of falls, study team members will ask about fall occurrences during each study visit, and the medical record will also be reviewed for any falls during the study period. Additional clinical secondary outcomes described will be collected from the electronic medical record. On postoperative day three, for patients not randomized to the family-support groups, the research team will ask family members about cumulative time spent with patients, and any interactive activities performed, during the first three postoperative days for comparison to family-based intervention groups.

Finally, research data recorded on paper will be stored in participant charts that will be located in locked cabinets in the Department of Anesthesiology at Michigan Medicine. Electronic data will be de-identified and stored online using the REDCap electronic research database, which resides on a secured, password-protected network managed by the Michigan Institute of Clinical and Health Research.

### Statistical analysis

***Sample size and power.*** Given its fluctuating and recurrent nature, delirium presence will be primarily assessed over time with logistic generalized estimating equation models as we have done previously ^36^. In brief, time and group will serve as fixed factors, and a group by time interaction term will be included. Interaction terms will be removed from models if no significant interaction effect is observed. These models allow for longitudinal data analysis in the setting of incomplete and missing data. Group models will be constructed individually with the control group serving as a reference, and an intention-to-treat approach will be followed. Power calculations were then conducted with generalized estimating equations by pooling intervention groups together for each calculation. For example, for detecting effects specific to the HELP intervention, the HELP arm (n=15) and combined arm (n=15) were pooled together and compared to the control arm (n=15) and family-support only arm (n=15) in a repeated-measures design with the binary outcome of delirium. Accounting for six equally spaced measurements (twice daily delirium assessments for the first three postoperative days), with an autoregressive correlation structure (baseline correlation 0.3) and linear missing data structure, a total sample size of 60 patients (n=30 in each intervention group) will provide >80% power to detect a difference in proportions of 15% (approximate Cohen’s effect size difference of 0.9) for experiencing an episode of delirium between groups, assuming a baseline proportion of 20% in the control group, with α=0.05. Power analysis was conducted using PASS 16 (PASS 2019 Power Analysis and Sample Size Software [2019]. NCSS, LLC. Kaysville, Utah, USA, ncss.com/software/pass). As an exploratory analysis, the interaction between HELP- and family-based support groups will be assessed using a generalized estimating equations model.

Descriptive statistics will be reported for secondary and exploratory outcomes. Inferential statistics will be deferred given the small sample size and pilot nature of the trial. Rigorous statistical analysis will be deferred for planned, follow-up, large-scale investigation. However, inferential statistics will be reported for fidelity measures described previously (Outcomes – Protocol Fidelity Measures). For continuous data, the Shapiro-Wilk test will be used to assess for normal distribution, and either independent t-tests or the Mann-Whitney U-test will be used, as appropriate. For categorical data, chi-squared or Fisher’s exact test will be used, as appropriate. Cohen’s kappa will be used to assess agreement between research-based CAM delirium assessments and FAM-CAM assessments.

***Sensitivity analysis and missing data.*** Missing data are anticipated for multiple outcomes described in this study. For each HELP visit, cumulative therapeutic time is routinely logged, as are reasons for deferred visits. Thus, the proportion of deferred shift visits, compared to all available shifts (excluding shifts missed due to early discharge) will be reported along with associated reasons ( Table 3). Nine total visits are anticipated during the first three postoperative days – daily morning, afternoon, and evening sleep visits. Similarly, missing CAM and FAM-CAM data are expected as well. Reasons for missing assessments will be presented in conjunction with barriers that family members and caretakers report for conducting FAM-CAM assessments. Given the relatively small sample size and pilot nature of this trial, imputation will be deferred for missing data.

**Table 3.** Anticipated reasons for missing data.

HELP Program – Reasons for deferred visits	Family and Caregivers – Reasons fo deferred FAM-CAM completion
HELP staff unavailable	Family unavailable for assessment
Patient engaged with other clinical staff	Patient engaged with other clinical staff
Undergoing medical testing or procedure	Undergoing medical testing or procedure
Visitors present	Not comfortable with performing evaluation
Patient sleeping	Patient sleeping
Patient declines visit	Patient refusal
Early discharge	Early discharge
Other	Other
Not specified	Not specified

HELP, Hospital Elder Life Program; FAM-CAM, Family Confusion Assessment Method.

### Pre-specified secondary and exploratory analyses

As described, a complementary line of analysis will focus on facilitators and barriers to implementing therapeutic protocols described, both from HELP- and family-based perspectives. Results will be used to inform therapeutic protocol design for a larger, follow-up trial. Descriptive reporting, based on mixed methods and Likert scale survey methodology ^29, 31^, will also be used to report experiences with clinical decision support systems. This sub-study analysis involving HELP staff members has received exemption from the University of Michigan Institutional Review Board (HUM00166883).

As a final exploratory line of analysis, an additional objective will be to test collect pilot data for testing differences in microbial patterns in delirious (vs. non-delirious) patients. Preliminary data suggest that colonization of multidrug-resistant organisms is common in older, hospitalized patients; furthermore, inpatient wards commonly harbor such organisms. The attendant complications of such colonization, particularly with respect to neurocognitive and clinical recovery, remain unknown. These microbial patterns will thus be tracked in enrolled patients. Trained study coordinators will collect samples from the hands and nares of enrolled patients using a culture swab during baseline enrollment, on the first postoperative morning (prior to interventions), and again on the morning of the second postoperative day. Study coordinators will also collect samples from 5 high-touch surfaces in patient’s rooms (e.g., bed controls/rail, call button/TV remote, tray table, phone, and toilet seat/commode) using a culture swab. These samples will be plated onto Bile Esculin Agar containing 6 g/mL vancomycin, Mannitol Salt Agar, and MacConkey Agar, and assessed for the presence of methicillin-resistant Staphylococcus aureus, vancomycin-resistant Enterococcus, and resistant gram-negative bacilli utilizing standard microbiology testing methods previously described ^37^.

### Pragmatic-explanatory trial continuum analysis

The RADAR Trial interventions were designed with intentions for high generalizability. HELP is now present at more than 200 hospital systems worldwide, and decision-support systems may help triage and organize support operations across such sites, particularly for those limited by personnel and/or resources. Alternatively, for hospitals without HELP, this trial will also assess the feasibility and efficacy of similar interventions administered by family members and caretakers.

**Figure 2.**
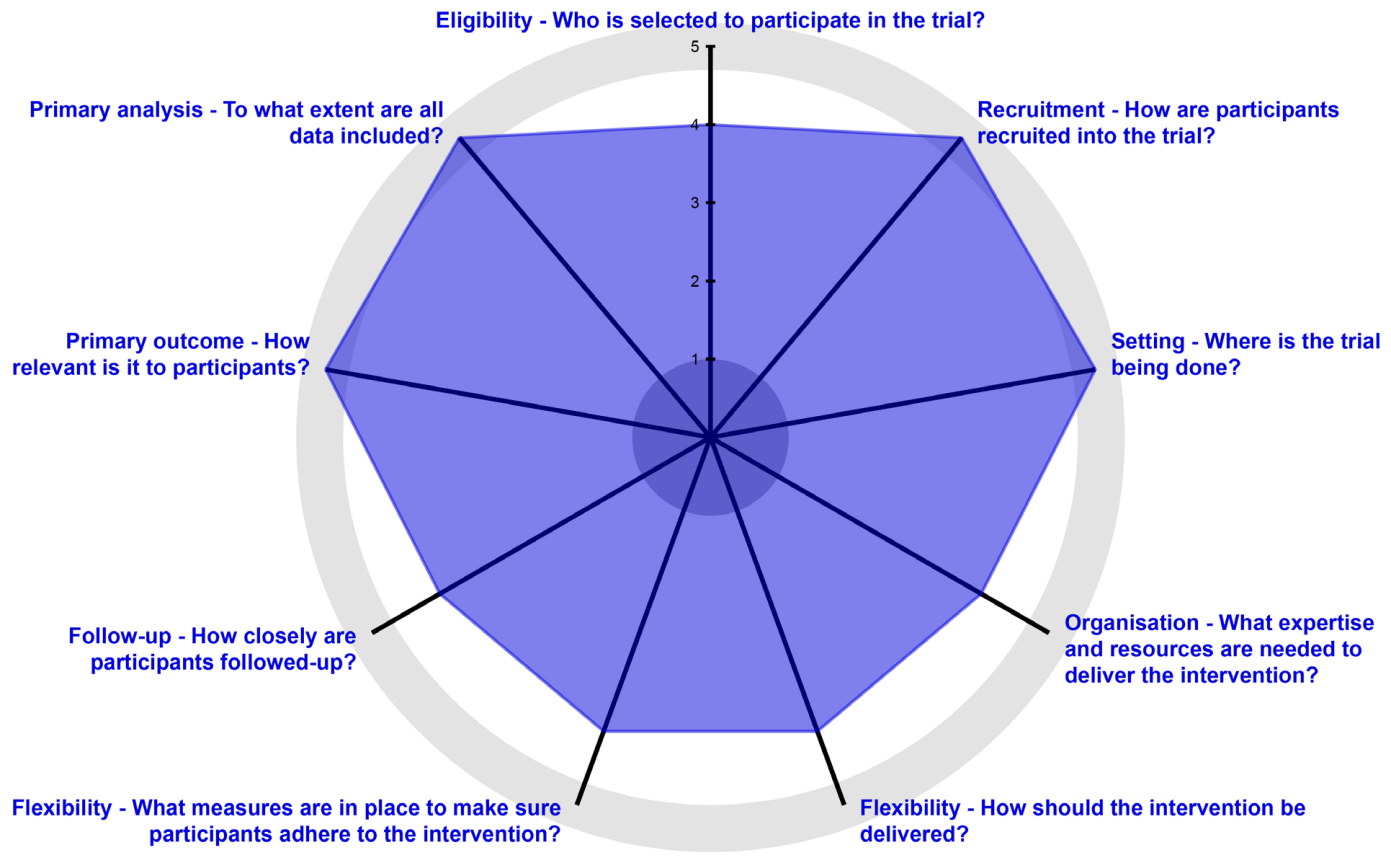
Pragmatic Explanatory Continuum Indicator Summary (PRECIS)-2 ^38^ tool depicting where RADAR resides on the pragmatic-explanatory trial continuum. After reviewing training materials, members of the RADAR team independently scored each of the 9 domains included in the PRECIS-2 Toolkit. For each domain, scores range from 1 to 5, with lower scores reflecting an explanatory nature, and higher scores reflecting pragmatic characteristics. Median scores are presented from all team members (n=10) that completed the PRECIS-2 toolkit scoring. The median score for each domain was either a 4 or 5, reflecting a fairly pragmatic study design overall.

To further study the pragmatic and explanatory elements of RADAR, trial members completed the PRagmatic-Explanatory Continuum Indicator Summary (PRECIS-2) toolkit ^38^. This is an assessment tool that characterizes the pragmatic and explanatory elements of clinical trial design, and the results inform as to where trial design resides on the pragmatic-explanatory continuum. For PRECIS-2 assessment, nine study domains are analyzed: eligibility criteria, recruitment, setting, intervention organization, flexibility of intervention delivery, flexibility of adherence, follow up, primary outcome, and primary analysis. Raters score each domain on a scale of 1 – 5, with lower scores reflecting explanatory trial characteristics, and higher scores suggesting a more pragmatic nature. RADAR Trial members independently scored each domain using the associated instructions, and median scores are illustrated in Figure 2. Each domain received a median score of 4 or 5, reflecting a relatively pragmatic study design. Regarding (1) eligibility criteria, the trial will recruit a heterogeneous, well-rounded group of surgical patients that will receive interventions similar to those administered postoperatively. There will be some exclusions based on family and caretaker availability, cognitive function, and surgical subtype. Recruitment (2) will be conducted at regularly scheduled preoperative clinic appointments, which occur as part of routine, standard care. The setting (3) The setting will be identical to where patients otherwise receive perioperative care. Regarding (4) the expertise and resources needed to deliver the interventions, the HELP-based interventions are closely related to usual care, though they may occur sooner and more thoroughly with the pager alerting system. However, there will be some additional resources and training required, particularly for family-based interventions. For (5) flexibility of intervention delivery, pager alerts can be reliably delivered and modified as needed. There are also many opportunities throughout the day to implement clinical protocols as outlined. However, to demonstrate effectiveness, the proposed interventions likely need to occur consistently and with adherence to the protocol. In terms of flexibility adherence (6), daily pager alerts will be reliably and automatically sent to HELP and family members to enhance protocol fidelity. Checklists will also be made available to family members. Follow-up (7) for most outcomes and operations will occur in the immediate postoperative period, and many outcomes described are obtainable via chart review. However, certain follow-up measures (e.g., CAM, 30-day surveys) require prospective collection from research team members, though raters did not raters generally did not anticipate this to be particularly burdensome or prohibitive. Delirium is the primary outcome (8), which is a common and serious postoperative outcome that is relevant to surgical patients. Lastly, the primary analysis plan (9) follows an intention-to-treat approach with longitudinal modeling that accounts for missing data. Although raters generally scored the trial design as pragmatic, raters were part of the trial team, and thus not independent assessors. This may introduce bias with regards to objectively rating explanatory and pragmatic elements of a trial ^39^.

### Data and safety monitoring plan

All participants will be monitored throughout the entire perioperative course by both the research team (including direct oversight by the PI) and clinical teams per standard care. The research team will monitor for adverse events, which will be reported per IRB guidelines. Participants will also have phone and pager numbers to the study coordinator and study PIs, and they are encouraged to contact our study team with any concerns that arise. While admitted to the hospital, participants will otherwise undergo routine monitoring and management per standard clinical practice. There will otherwise be no additional data management committee, and no interim analyses or audits are planned for this trial. For data storage, primary source paper documents will be stored in locked files within the Department of Anesthesiology at Michigan Medicine. Electronic data will be de-identified and entered into the online REDCap, database, which is managed by the Michigan Institute of Clinical and Health Research Management Core. Lastly, all protocols and consent forms approved by the University of Michigan Medical School IRB are reviewed yearly.

### Strengths and limitations

Multiple strengths of this trial are worth noting. First, trial design is relatively pragmatic, as perioperative care will be minimally altered. The HELP-based activities described already take place at Michigan Medicine; a single page will be sent to HELP to assist with triage and focus therapeutic activity on relatively high-risk patients. The system can conceivably benefit any patient regardless of surgical subspecialty, as supportive protocols outlined could be implemented – or adapted – as part of enhanced recovery protocols irrespective of surgical service. The study also offers an innovative approach to integrating family members and caretakers in the postoperative recovery process. Preliminary data suggest that family involvement is both feasible ^17^ and may improve clinical recovery after major surgery ^18^. Thus, both HELP- and family-based support systems offered might provide an effective, practical approach to mitigating delirium risk while minimizing strain to the healthcare system.

Considerable limitations are also worth discussion. The statistical powering strategy crucially assumes there is no interaction between the interventions (e.g., HELP- and family-based support), which may not be the case. However, this pilot phase will help generate effect size and feasibility data for refining future protocols and power calculations. Next, both HELP officials and family members may be subject to the Hawthorne effect ^40^. That is, individuals may modify their behavior under study conditions. Both HELP officials and family members may rigorously perform study protocols knowing that performance is being monitored. As such, protocol effectiveness will likely decrease in non-research settings. For the HELP program, performance measures will be compared to historical controls (2018 HELP records) to assess for this effect. Family members of patients not randomized to family-based support interventions may still elect to spend more time with patients after learning about the trial and proceeding with enrollment. To assess for latent family support in the control groups, the research team will ask family members about time and activities with patients for comparisons to structured family-based support allocation groups. This will be assessed on the afternoon of postoperative day three, at the end of the inpatient study window, to avoid inadvertent introduction of study-related family support interventions. Participants who are able, and motivated, to engage with HELP- and family-based support may inherently be less prone to delirium compared to those who do not adhere to study protocols. Additionally, while family members will receive basic training and education on FAM-CAM administration, they will not receive rigorous training for assessing reliability or accuracy. Such training could be pursued in a follow-up trial. Lastly, only patients with family members and/or caretakers – who will be available for the first three days of hospitalization – will be eligible for the trial. Thus, patients without such social support will be ineligible. These eligibility criteria thus exclude a group of patients who may be particularly vulnerable to delirium (i.e., less social support) ^41^ and reduce trial pragmatism.

### Ethical considerations

Emanuel *et al*. ^42^ have proposed seven universal requirements, drawn from landmark codes and declarations, for comprehensively incorporating all relevant ethical considerations for clinical research, particularly in the context of aiming to improve health and/or increase understanding of human biology. These considerations are presented in question format, along with responses for this trial, in Table 4.

**Table 4.** Ethical Considerations.

**1. What scientific or social value will be gained from the proposed research?**
The proposed clinical support system may improve neurocognitive and clinical recovery for older, vulnerable surgical patients. Given the common occurrence of delirium in this population, along with related complications (e.g., falls, delayed discharge), postoperative clinical complications may threaten the health and functional independence of older surgical patients. By aiming to provide therapeutic, supportive activity early (and frequently) during postoperative recovery, these clinical support systems may reduce the risk of delirium and associated outcomes. As such, the proposed intervention has the potential for improving health and well-being for such vulnerable patients.
**2. Will accepted scientific principles and methods be used to produce reliable and valid data?**
This trial incorporates multiple strategies for rigorous data acquisition and analysis. Prospective, block-stratified randomization will occur after trial enrollment. The randomized approach will mitigate selection bias during the allocation process and increase the likelihood that findings observed are attributable to the intervention. The sensitivity analysis will also provide transparency regarding missing data and challenges related to intervention fidelity. Lastly, there is a possibility of observer bias (i.e., Hawthorne effect) with HELP staff and family members, as behavior may be modified given the known presence of an ongoing trial. As such, a historical control group will be included from 2018 for determining HELP measures prior to trial initiation. Ongoing family support data will also be collected from groups not randomized to family-based support interventions.
**3. Are participants selected such that stigmatized and vulnerable individuals are not targeted for risky research, and** **socially affluent and powerful are not targeted for beneficial research?**
As outlined in the eligibility criteria, all surgical patients (≥70 years old) at high risk for postoperative complications will be eligible for enrollment, regardless of demographic or social background. In a preliminary study that predicted postoperative risk of complications in older patients, those who were ≥70 years of age presenting for major surgery had a seven-fold increased risk of major complications compared to minor surgery ^24^. Thus, this study specifically aims to include this vulnerable demographic of patients for beneficial research.
**4. Is there a favorable benefit-to-risk ratio for participants? Will the benefits to the participant, and/or society,** **outweigh any potential risk to the enrolled participants?**
The risks associated with this study are minimal. Those randomized to the control group will receive standard perioperative care. If randomized to an interventional group, the patients will likely receive enhanced support from HELP staff and/or family members. Risks associated with these interventions are minimal, but may include anxiety and fatigue from cognitive and functional interventions to improve health after surgery. We feel that trial benefits outweigh these risks, particularly if the intervention reduces the risk of delirium and possible downstream consequences (e.g., falls, delayed discharge).
**5. Will independent reviews take place such that a committee, with an appropriate range of expertise, will have the** **ability to approve, amend, or terminate the study?**
The trial has been approved by the University of Michigan Medical School Institutional Review Board, and annual reviews will occur per institutional protocols. The study team will monitor for Adverse Events and Other Reportable Information or Occurrences in compliance with Institutional Review Board protocols.
**6. Will informed consent be obtained from all participants prior to enrollment?**
Written informed consent will be obtained from all participants prior to trial enrollment. Consent forms are written in conjunction with Institutional Review Board requirements, which require discussion of the following: purpose of the study, participant eligibility, study procedures, information about risks and benefits, ending participation, financial considerations, confidentiality, and study team contact information.
**7. Does the proposed study engender respect for potential and enrolled subjects?**
Patients will be free and able to withdraw from the trial at any time, and several measures will be taken to maintain participant privacy and confidentiality. If new, unanticipated risks or benefits become apparent during the course of the trial, the protocol will be amended and participants will be made aware of any new risks or benefits of study inclusion. Participant welfare will be respected and maintained throughout trial operations. Adverse events will be reported per Institutional Review Board guidelines, and clinical care will otherwise proceed per perioperative standards at Michigan Medicine.

## Dissemination

The trial will be presented at academic conferences, presentations, and medical journals. As mentioned, the trial was registered on www.clinicaltrials.gov ( NCT04007523), and any protocol changes will be made publicly available on this registry. This manuscript currently reflects the 4 ^th^ version of the protocol (August 21 ^st^, 2020).

If the results demonstrate improved HELP evaluation and therapeutic practices, the paging-based support intervention will be tested in a large-scale trial to assess effectiveness for reducing delirium incidence and related consequences. Family-based interventions may be included as well depending on success and feasibility with family-led delirium screening and prevention procedures described. The nature of such future interventions may be modified depending on survey results from HELP personnel and family members.

## Conclusions

Delirium remains a pressing public health issue, and associated consequences bear significant morbidity. The proposed clinical decision support system has the potential to improve the environment for neurocognitive and clinical recovery for high-risk patients. The paging support system is also relatively pragmatic, and if successful, could be used across various healthcare systems and tailored accordingly. If encouraging preliminary results are demonstrated, the proposed interventions will be tested in a large-scale trial for clinical effectiveness.

## Data availability

### Underlying data

No data are associated with this article

### Extended data

Open Science Framework: Recommendations and Alerting for Delirium Alleviation in Real-Time (RADAR): Extended Data. https://doi.org/10.17605/OSF.IO/UYZ92 ^23^.

This project contains the following extended data:

- RADAR Family Consent v1.0 7-23-2019.pdf
- RADAR Subject Consent v1.0 7-23-2019.pdf
- HELP Focus Group Discussion Guide.pdf
- RADAR – HELP Facilitators and Barriers Survey.pdf
- RADAR Family Survey – Facilitators and Barriers.pdf

These data are available under the terms of the Creative Commons Zero "No rights reserved" data waiver (CC0 1.0 Public domain dedication).

Data collection forms related to the Hospital Elder Life Program (e.g., Confusion Assessment Method, Family Confusion Assessment Methods) are subject to copyright restriction and are available on the Hospital Elder Life Program Website ( https://www.hospitalelderlifeprogram.org/). The Hospital Anxiety and Depression Scale is also subject to copyright restriction and can be accessed at the following website: https://www.gl-assessment.co.uk/. The 36-Item Short Form Survey (SF-36) can be found on the Rand Healthcare website ( https://www.rand.org/health-care/surveys_tools/mos/36-item-short-form.html), and the PROMIS cognitive function assessments can also be found on the associated website ( http://www.healthmeasures.net/explore-measurement-systems/promis).

### Reporting guidelines

Open Science Framework: CONSORT Pilot and SPIRIT checklists, and WHO Trial Registration Data Set for ‘Recommendations and Alerting for Delirium Alleviation in Real-Time (RADAR): Protocol for a pilot randomized controlled trial’. https://doi.org/10.17605/OSF.IO/UYZ92 ^23^.

- RADAR CONSORT Pilot Checklist.doc
- RADAR SPIRIT Checklist.doc
- RADAR WHO Trial Registration Data Set.docx

Reporting guidelines are available under the terms of the Creative Commons Zero "No rights reserved" data waiver (CC0 1.0 Public domain dedication).
