# Supplementary material for: Recommendations and Alerting for Delirium Alleviation in Real-Time (RADAR): Protocol for a pilot randomized controlled trial
Source: F1000Res. 2020 Sep 1;8:1683. Originally published 2019 Sep 24. [Version 2] doi: 10.12688/f1000research.20597.2 (PMC7471625; doi:10.12688/f1000research.20597.2)
Supplement: Peer review history

# Peer review: Reviewer response for version 2

**DOI:** 10.5256/f1000research.29050.r70561

**Published:** 2020-09-07

**Authors:** Nicolai Goettel

**Affiliations:** 1 Department of Anesthesia, Prehospital Emergency Medicine and Pain Therapy, University Hospital Basel, University of Basel, Basel, Switzerland; 2 Department of Clinical Research, University of Basel, Basel, Switzerland

The authors have addressed all comments and suggestions in this revised version. Good luck with the trial!

Is the study design appropriate for the research question?

Yes

Is the rationale for, and objectives of, the study clearly described?

Yes

Are sufficient details of the methods provided to allow replication by others?

Yes

Are the datasets clearly presented in a useable and accessible format?

Not applicable

Reviewer Expertise:

Delirium, postoperative neurocognitive disorders

I confirm that I have read this submission and believe that I have an appropriate level of expertise to confirm that it is of an acceptable scientific standard.

# Peer review: Reviewer response for version 2

**DOI:** 10.5256/f1000research.29050.r70562

**Published:** 2020-09-03

**Authors:** Bala Subramaniam

**Affiliations:** 1 Department of Anesthesiology, Harvard Medical School, Boston, MA, USA

The authors have addressed my concern. I have no reservations

Is the study design appropriate for the research question?

Partly

Is the rationale for, and objectives of, the study clearly described?

Yes

Are sufficient details of the methods provided to allow replication by others?

Yes

Are the datasets clearly presented in a useable and accessible format?

Not applicable

Reviewer Expertise:

Postoperative delirium, Perioperative hemodynamics, Resilience

I confirm that I have read this submission and believe that I have an appropriate level of expertise to confirm that it is of an acceptable scientific standard.

# Peer review: Reviewer response for version 1

**DOI:** 10.5256/f1000research.22648.r69809

**Published:** 2020-08-20

**Authors:** Nicolai Goettel

**Affiliations:** 1 Department of Anesthesia, Prehospital Emergency Medicine and Pain Therapy, University Hospital Basel, University of Basel, Basel, Switzerland; 2 Department of Clinical Research, University of Basel, Basel, Switzerland

Thank you for the opportunity to review this work.

 Vlisides and coworkers present the protocol for a pilot randomized controlled trial (RCT) investigating the effect of a multicomponent decision support system, which sends automated alerts and recommendations to patient-care programs and family members for high-risk patients, on the incidence of postoperative delirium.

 High-risk, non-cardiac surgery patients (≥70 years old) are allocated to 4 groups: (1) usual care group (n=15), (2) Hospital Elder Life Program (HELP)-based paging system (n=15), (3) family-based paging system (n=15), and (4) combined HELP- and family-based system (n=15).

 Primary outcome measure is the incidence of postoperative delirium assessed by Confusion Assessment Method (CAM) screening. Secondary outcome measures are diverse performance metrics for postoperative neurocognitive disorders and clinical recovery.

 The study protocol is methodologically sound and closely follows the CONSORT and SPIRIT guidelines.

 Even though patients are randomly allocated to treatment groups, there is a risk of apprehension bias and the Hawthorne effect. Study participants, especially those in groups 2–4, may respond differently due to being observed, or individuals may modify an aspect of their behavior in response to their awareness of being observed.

 There is also a considerable risk of compliance bias. Preventive interventions for postoperative neurocognitive disorders tend to be rather complex and time-consuming for all persons involved. Participants compliant with the intervention may differ in some way from those not compliant, which can systematically affect the outcome of interest. In this context, one could argue that higher-functioning patients are more compliant with the intervention; hence, they may present less postoperative delirium.

 Figure 1 (CONSORT flow chart): There seems to be a two-step group allocation rather than the 1:1:1:1 group allocation mentioned in the text. What is the difference between “usual HELP care” and “enhanced HELP system”?

 One eligibility criterion for recruitment is a high risk for postoperative delirium. However, the inclusion criteria only partially reflect a high-risk surgical population. In other words, being 70 years of age and older and undergoing major non-cardiac, non-intracranial, and non-major vascular surgery might not be sufficient to reach a high-risk level for postoperative delirium. I would suggest to extend the inclusion criteria to participants with an even higher risk of postoperative neurocognitive disorders, such as patients with pre-existing cognitive impairment.

 Given the above, the overall incidence of postoperative delirium (or other postoperative neurocognitive disorders) could potentially be quite low in the studied population, or at least not attain the 20% in the control group assumed by the authors. This would subsequently lead to statistically weak results (underpowered study).

 In summary, this research is hypothesis-driven and the study is well-designed. I comment the authors for making the effort to include most (if not all) state-of-the-art nonpharmacological interventions to prevent postoperative neurocognitive disorders in a single RCT. I wish the investigators best of luck with the ongoing trial.

Is the study design appropriate for the research question?

Yes

Is the rationale for, and objectives of, the study clearly described?

Yes

Are sufficient details of the methods provided to allow replication by others?

Yes

Are the datasets clearly presented in a useable and accessible format?

Not applicable

Reviewer Expertise:

Delirium, postoperative neurocognitive disorders

I confirm that I have read this submission and believe that I have an appropriate level of expertise to confirm that it is of an acceptable scientific standard.

# Response

**Published:** 2020-08-21

**Authors:** Phillip Vlisides

**Affiliations:** University of Michigan Medical School, USA

Thank you for the opportunity to review this work.

Vlisides and coworkers present the protocol for a pilot randomized controlled trial (RCT) investigating the effect of a multicomponent decision support system, which sends automated alerts and recommendations to patient-care programs and family members for high-risk patients, on the incidence of postoperative delirium.

High-risk, non-cardiac surgery patients (≥70 years old) are allocated to 4 groups: (1) usual care group (n=15), (2) Hospital Elder Life Program (HELP)-based paging system (n=15), (3) family-based paging system (n=15), and (4) combined HELP- and family-based system (n=15).

Primary outcome measure is the incidence of postoperative delirium assessed by Confusion Assessment Method (CAM) screening. Secondary outcome measures are diverse performance metrics for postoperative neurocognitive disorders and clinical recovery.

The study protocol is methodologically sound and closely follows the CONSORT and SPIRIT guidelines.

Even though patients are randomly allocated to treatment groups, there is a risk of apprehension bias and the Hawthorne effect. Study participants, especially those in groups 2–4, may respond differently due to being observed, or individuals may modify an aspect of their behavior in response to their awareness of being observed.

**RESPONSE: We would like to thank the reviewer for the thorough, thoughtful review. We agree that apprehension bias, and the Hawthorne effect, are real possibilities with this trial. We have attempted to mitigate this bias with the following strategies (as outlined in the Strength and Limitations section):**

**First, we will compare HELP metrics (specifically, time to initial evaluation and cumulative time spent with patients) with this trial compared to historical controls (2018 records). If performance measures are improved in the control group compared to the 2018 historical controls, this suggests that the Hawthorne effect may be present. If performance is similar, this would weight against the presence of the Hawthorne effect.**

**Second, we will assess cumulative time family members spent with patients, along with any potential therapeutic activities performed, even for patients/families not randomized to family-based support interventions. We will wait to collect these data until postoperative day three, so as to not introduce the idea of providing enhanced family support during the first three postoperative days.**

There is also a considerable risk of compliance bias. Preventive interventions for postoperative neurocognitive disorders tend to be rather complex and time-consuming for all persons involved. Participants compliant with the intervention may differ in some way from those not compliant, which can systematically affect the outcome of interest. In this context, one could argue that higher-functioning patients are more compliant with the intervention; hence, they may present less postoperative delirium.

**RESPONSE**: **We agree that this limitation is present. Indeed, patients who are able – and motivated – to engage with HELP- and family-based support may inherently be less prone to postoperative delirium and related neurocognitive disorders. We have added this limitation to the protocol manuscript.**

Figure 1 (CONSORT flow chart): There seems to be a two-step group allocation rather than the 1:1:1:1 group allocation mentioned in the text. What is the difference between “usual HELP care” and “enhanced HELP system”?

**RESPONSE**: **The reviewer is correct. This two-step allocation helps to facilitate our randomization procedures, but the final 1:1:1:1 allocation remains the same.**

**At our institution, with usual HELP care, the team manually reviews the census of surgical wards daily and then sees various patients throughout the day based on volunteer availability and visitation patterns. There is presently no structured triage system for prioritizing high risk patients, as outlined in the manuscript. As a result, some high risk patients may not be seen until the second or third postoperative day, as discussed in the Introduction section. This information has been included in the Interventions section of the protocol manuscript.**

One eligibility criterion for recruitment is a high risk for postoperative delirium. However, the inclusion criteria only partially reflect a high-risk surgical population. In other words, being 70 years of age and older and undergoing major non-cardiac, non-intracranial, and non-major vascular surgery might not be sufficient to reach a high-risk level for postoperative delirium. I would suggest to extend the inclusion criteria to participants with an even higher risk of postoperative neurocognitive disorders, such as patients with pre-existing cognitive impairment.

**RESPONSE: We agree with the reviewer that there exists a higher-risk tier, which includes patients with pre-existing cognitive impairment (along with other risk factors). Data from a frailty prediction model for surgical patients ≥70 years old, developed by our institution, suggest that high surgical complexity alone (i.e., procedure requiring considerable perioperative resources with inpatient admission) was associated with increased risk of geriatric complications, including delirium and related outcomes (AOR 12.1, 95% CI: 6.4 – 22.7; p<0.001) (Min et al., JAMA Surg. 2017 Dec; 152(12): 1126–1133). As such, for a feasibility/pilot trial, we surmised that this population would be appropriate for testing both efficacy and feasibility with such candidate interventions. Focusing on patients with prior neurocognitive impairment may be more challenging logistically, specifically with regards to determining basic barriers to implementation. However, we will certainly consider this population in the future as we continue to determine the impact and barriers with respect to such decision-support systems.**

Given the above, the overall incidence of postoperative delirium (or other postoperative neurocognitive disorders) could potentially be quite low in the studied population, or at least not attain the 20% in the control group assumed by the authors. This would subsequently lead to statistically weak results (underpowered study).

**RESPONSE: Indeed, this is an important consideration with postoperative delirium. Prior trials at our institution have revealed an incidence of approximately 20% with such non-cardiac surgery populations (Avidan et al., Lancet 2017; Vlisides et al., JNA 2019). In fact, the inclusion criteria for these prior trials included patients >60 years of age. As such, we anticipate that this 20% estimate will be accurate. Nonetheless, if the incidence differs significantly in this pilot trial, these results will better inform future power calculations for subsequent trials.**

In summary, this research is hypothesis-driven and the study is well-designed. I comment the authors for making the effort to include most (if not all) state-of-the-art nonpharmacological interventions to prevent postoperative neurocognitive disorders in a single RCT. I wish the investigators best of luck with the ongoing trial.

**RESPONSE: We would like to thank the reviewer for the constructive, encouraging feedback.**

# Peer review: Reviewer response for version 1

**DOI:** 10.5256/f1000research.22648.r56796

**Published:** 2019-12-17

**Authors:** Bala Subramaniam

**Affiliations:** 1 Department of Anesthesiology, Harvard Medical School, Boston, MA, USA

The authors have designed an elegant study to  maximize their ability to use existing supportive interventions in the prevention of delirium. This study is very much needed and wish the very best for the authors to complete all the elements proposed in this study.

1. The authors note this as a factorial design. In Fig. 1 this is categorized as usual HELP vs. Enhanced HELP system. The study is powered for interventions vs. controls.  Enhanced HELP, Family support and HELP plus family support are all intervention groups. Is this powered to 15 vs. 45 (unbalanced)? While they note that n=30 in their power calculations. This is confusing.  
2. Given 1, is factorial design the best suited for this study?  
3. In the HELP group, all efforts are taken to make sure the investigators get two assessment right before they become eligible to score CAM. Why aren't the same efforts taken for FAMCAM as well? I suppose there will be logical issues, perhaps scoring with case videos might be one option.
4. Inclusion criteria mentions high-risk group and yet age is the only one chosen to define high risk. Or have I missed other risk factors in the inclusion criteria?
5. Family support can do certain things and HELP can do certain things. I presume this will be complimentary. It will be interesting to find out the results of this study.

Is the study design appropriate for the research question?

Partly

Is the rationale for, and objectives of, the study clearly described?

Yes

Are sufficient details of the methods provided to allow replication by others?

Yes

Are the datasets clearly presented in a useable and accessible format?

Not applicable

Reviewer Expertise:

Postoperative delirium, Perioperative hemodynamics, Resilience

I confirm that I have read this submission and believe that I have an appropriate level of expertise to confirm that it is of an acceptable scientific standard, however I have significant reservations, as outlined above.

# Response

**Published:** 2020-08-21

**Authors:** Phillip Vlisides

**Affiliations:** University of Michigan Medical School, USA

The authors have designed an elegant study to maximize their ability to use existing supportive interventions in the prevention of delirium. This study is very much needed and wish the very best for the authors to complete all the elements proposed in this study.

 The authors note this as a factorial design. In Fig. 1 this is categorized as usual HELP vs. Enhanced HELP system. The study is powered for interventions vs. controls.  Enhanced HELP, Family support and HELP plus family support are all intervention groups. Is this powered to 15 vs. 45 (unbalanced)? While they note that n=30 in their power calculations. This is confusing.

**RESPONSE:** **We would like to thank the reviewer for the thorough and thoughtful review. We can certainly understand the confusion regarding power/sample size calculations and will attempt to clarify.**

**The power calculations pool two intervention groups together for each calculation. For example, for detecting effects specific to the HELP intervention, the HELP arm (n=15) and combined arm (n=15) were pooled together and compared to the control arm (n=15) and family-support only arm (n=15). This strategy was also used for the family-specific intervention calculations. This is a conventional strategy for factorial trials described by Montgomery et al. (BMC Med Res Methodol. 2003; 3:26). We have added this language to the Statistical Analysis section of the manuscript.**

**We acknowledge that his powering strategy has shortcomings. For example, this strategy crucially assumes there is no interaction between the interventions (e.g., HELP- and family-based support), which may not be the case in this trial. However, in the context of a pilot clinical trial for generating effect size data, testing feasibility, and informing future trial protocols, we surmised that this would be a reasonable initial strategy. We have added this limitation (and additional explanation) to the protocol manuscript.**

 Given 1, is factorial design the best suited for this study?

**RESPONSE: As discussed above, there are indeed methodological limitations with this approach. The main objective of this pilot study, however, is to determine both efficacy and feasibility with these decision-support systems for both HELP- and family-based support. The factorial design allows for separate (and combined) analyses with respect to each intervention arm. This is particularly salient given that many hospital systems do not have HELP, and such family-based support systems might be alternative supportive care options for such hospital systems. In fact, the results from this study might inform future design for separate trials (one specific to hospitals with HELP, and a separate trial for hospitals without HELP). The factorial design allows us to (1) efficiently collect initial data on these interventions simultaneously and (2) analyze effects both separately and jointly via exploratory interaction analysis.**

 In the HELP group, all efforts are taken to make sure the investigators get two assessment right before they become eligible to score CAM. Why aren't the same efforts taken for FAMCAM as well? I suppose there will be logical issues, perhaps scoring with case videos might be one option.

**RESPONSE: The reviewer raises important points about CAM and FAM-CAM methodology. Logistical issues are indeed a concern. Family members will only be scoring their own family members, and there will be a limited number of assessments (i.e., three days) prior to discharge. Thus, training opportunities would be limited. This is nonetheless an important methodological issue. We did not incorporate case videos for family members, but this could be considered for future protocols. We have added this as a study limitation in the protocol manuscript.**

 Inclusion criteria mentions high-risk group and yet age is the only one chosen to define high risk. Or have I missed other risk factors in the inclusion criteria?

**RESPONSE: The high-risk label is derived from one of our group’s prior studies, in which patients at least 70 years of age, presenting for surgery with work-related value units suggestive of high surgical complexity were among the highest risk of perioperative complications (including delirium) regardless of comorbidity burden (JAMA Surg. 2017 152(12): 1126-1133). Patients 70 years of age presenting for major surgery were assigned nine points in the screening tool published (JAMA Surg. 2017 152(12): 1126-1133), and a score** **³** **9 points was proposed as the optimal cutoff for predicting major perioperative complications (e.g., infection, delirium, falls). We have clarified this language in the updated protocol manuscript.**

 Family support can do certain things and HELP can do certain things. I presume this will be complimentary. It will be interesting to find out the results of this study.

**RESPONSE: The reviewer is correct – the HELP- and family-based activities will be complementary. In the future, these interventions may work in a combined, complementary manner, or they each may be suitable as an independent set of interventions. Family-based support protocols might, for example, be helpful for hospitals without HELP availability.**

**We otherwise thank the reviewer for the review and analysis of the protocol.**
